# IDIOPATHIC VULVAR CALCINOSIS: THE COUNTERPART OF IDIOPATHIC SCROTAL CALCINOSIS

**DOI:** 10.4103/0019-5154.43220

**Published:** 2008

**Authors:** Vandana Mehta, C Balachandran

**Affiliations:** *From the Departments of Skin and STD, Kasturba Medical College, Manipal, Karnataka, India. E-mail: vandanamht@yahoo.com*

Calcinosis cutis is characterized by the deposition of hydroxyapatite crystals of calcium phosphate in the skin. It is commonly encountered as a consequence of connective tissue disease or metabolic abnormalities but sometimes could be idiopathic. We report a case of idiopathic calcinosis cutis of the vulva in a healthy woman.

**Fig. 1:**
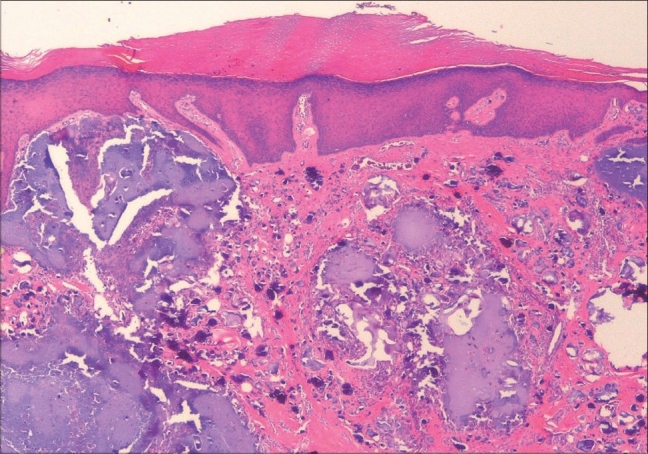
10× photomicrograph showing the amorphous calcific deposits in the dermis

A 35-year-old woman presented with multiple asymptomatic yellowish nodules on the external genitalia of one year duration. A few of the nodules broke down spontaneously discharging a chalky white material. She was otherwise healthy and had no systemic complaints. There was neither a history of trauma nor any inflammatory process in the vulvar skin prior to the development of lesions. The hematologic and biochemistry panel such as liver and renal function, serum calcium, phosphorous, electrolytes, uric acid and parathyroid hormone levels were normal. An excision biopsy of the nodule showed features suggestive of calcinosis cutis (Fig. 1).

Cutaneous calcification may be divided into four major categories: dystrophic, metastatic, iatrogenic and idiopathic.^1^ Though dystrophic calcinosis is fairly common, idiopathic calcification is rare, and usually no underlying cutaneous cause can be identified. It commonly presents as painless, firm papules and nodules that appear in childhood and adolescence and gradually increase in number and size. Lesions may be solitary or pedunculated and are initially skin-colored, but as they grow larger they become yellowish and lobulated breaking down spontaneously or when compressed to produce a chalky white material. Microscopic examination typically shows large granular deposits of deeply basophilic material which stains black with Von Kossa stain for calcium.^2^

The pathogenesis of idiopathic vulvar calcinosis is highly disputed. The principle debate concerning the cause is whether calcium is deposited at the site of inflamed epidermal cysts or whether the calcific nodules are truly idiopathic. Although several authors have suggested that vulvar calcinosis results from dystrophic calcification of the inflammed epidermal cysts, several others have refuted the above facts and found it to be truly idiopathic.^3^

Idiopathic calcinosis cutis of the vulva is rare with only seven cases reported worldwide. Besides, it has also been reported over the neck after rhytidectomy,^4^ the sctotal wall^5^, shaft of the penis^6^ and the areola of the breast.^7^ Cornelius *et al*, have postulated that tiny apatite crystals seen only on electron microscopy in cases of idiopathic calcinosis cutis might act as a source of injury leading to secondary deposition of acid mucopolysaccharides, which become a matrix for crystal formation and calcification. What initiates the deposition of these crystals is not yet known.^8^ In our case no predisposing cause for calcification could be identified and since the patient was asymptomatic and not keen on surgical removal the lesions were not excised.
